# Chemical composition and biological effects of *Lavandula angustifolia* Mill., essential oils

**DOI:** 10.1186/s13568-025-01971-w

**Published:** 2025-11-05

**Authors:** Oumayma Aguerd, Hamza Elhrech, Nasreddine El Omari, Taoufiq Benali, Mohamed Akhazzane, Mohammed Mostakim, Safae Ouma, Leila Khattabi, Mohammed Amanullah, Naoual El Menyiy, Rahul G. Ingle, Long Chiau Ming, Abdelhakim Bouyahya

**Affiliations:** 1 Laboratory of Human Pathologies Biology, Department of Biology, Faculty of Sciences, Mohammed V University in Rabat, 10106 Rabat, Morocco; 2 High Institute of Nursing Professions and Health Techniques of Tetouan, Tetouan, Morocco; 3 Laboratory of Ecotoxicology, Bioresources, and Coastal Geomorphology, Polydisciplinary Faculty of Safi, Cadi Ayyad University, PO Box 4162, 46000 Safi, Morocco; 4 Cité de L’innovation, Université Sidi Mohamed Ben Abdellah, Route Immouzer, P.O. Box 2626, 30000 Fez, Morocco; 5 Research Laboratory On Cancer and Chronic Diseases, Faculty of Medicine and Pharmacy Tangier, Abdelmalek Essaadi University, Tangier, Morocco; 6 Department of Clinical Biochemistry, College of Medicine, King Khalid University, Abha, Saudi Arabia; 7 Laboratory of Pharmacology, National Agency of Medicinal and Aromatic Plants, BP 159, 34000 Principal, Taounate, Morocco; 8 Datta Meghe College of Pharmacy, Datta Meghe Institute of Higher Education and Research (Deemed to be University), Sawangi, Wardha, India; 9 Faculty of Medical and Life Sciences, Sunway University, Sunway City, Malaysia

**Keywords:** Diabetes, *Lavandula angustifolia* mill., GC–MS analysis, Dermatoprotective effects, Neuroprotective effects, Molecular model

## Abstract

This study aimed to investigate the chemical composition and biological activities of *Lavandula angustifolia* essential oil (LAEO) cultivated in Ouezzane, Northwest Morocco. The chemical profile of LAEO was determined using gas chromatography–mass spectrometry (GC–MS), and its bioactivities, including antioxidant, antidiabetic, dermatoprotective, neuroprotective, and antibacterial properties, were evaluated through in vitro assays and molecular docking studies. GC–MS analysis identified 23 compounds, predominantly oxygenated monoterpenes, with linalyl acetate (31.78%) and linalool (16.58%) as major constituents. LAEO exhibited strong antioxidant activity in DPPH, ABTS, and ferric reducing power assays. It also displayed potent antibacterial effects, particularly against Gram-positive bacteria. For antidiabetic potential, LAEO demonstrated significant inhibition of α-amylase and α-glucosidase (IC_50_: 131.94 ± 1.82 and 86.20 ± 1.23 µg/mL, respectively). Dermatoprotective effects were observed through tyrosinase inhibition (IC_50_: 140.93 ± 3.30 µg/mL), while neuroprotective potential was evidenced by greater inhibition of acetylcholinesterase (AChE) than butyrylcholinesterase (BChE). These findings were further supported by in silico molecular docking analysis. In summary, LAEO presents a promising natural source of bioactive molecules with potential applications in food and pharmaceutical industries, addressing antioxidant, antidiabetic, and other therapeutic needs.

## Introduction

Oxidative stress, driven by reactive oxygen species (ROS) from internal and external sources, contributes to the progression of various diseases, including diabetes, neurodegenerative disorders like Alzheimer’s disease (AD), and skin damage (Benkhaira et al. 2023; National Agency of Medicinal and Aromatic Plants et al. 2023). In AD, ROS promote *β*-amyloid accumulation and neurofibrillary tangles (National Agency of Medicinal and Aromatic Plants et al. 2023), while in diabetes, they weaken antioxidant defenses, worsening the disease (Jeddi et al. 2023). ROS also cause protein glycation, lipid peroxidation, DNA damage, and enzyme inhibition, leading to inflammation and cellular dysfunction (Benkhaira et al. 2023). The skin, frequently exposed to UV radiation and environmental factors, is highly vulnerable to ROS-induced damage (Briganti and Picardo 2003). Additionally, the rise of drug-resistant pathogens complicates microbial disease treatment (Farhadi et al. 2019).

Natural molecules are an endless reservoir for the discovery of novel bioactive compounds, serving as the origin of numerous pharmaceuticals (Lahlou 2013). They offer a promising alternative to synthetic drugs, often featuring lower or non-existent toxicity, more affordable prices, and potentially comparable efficacy to their synthetic counterparts (Guaouguaou et al. 2018). Essential oils (EOs), synthesized by aromatic plants as secondary metabolites, are notable among natural substances for their complex molecular composition (Bouyahya et al. 2019). EOs are extracted from various plant parts, including leaves, flowers, stems, and roots. Their intricate molecular structure comprises a range of organic compounds, including terpenoids that contribute to their characteristic aromas, along with ketones, acids, aldehydes, esters, and alcohols (Angane et al. 2022). These compounds contribute to their distinctive aromas and confer a range of therapeutic properties, such as antimicrobial, anti-inflammatory, antioxidant, and neuroprotective effects (Benkhaira et al. 2023). Today, EOs derived from various aromatic plants and their derivatives are extensively studied for their multifaceted applications beyond their traditional roles as raw materials. They find applications in various industries, including pharmaceuticals, agriculture, food, health, cosmetics, and perfumery (Al-Mijalli et al. 2022).

The Lamiaceae family is a major source of polyphenols and possesses numerous pharmacological properties documented in the literature. *Lavandula angustifolia*, a member of this family, originates from the mountainous regions of the Mediterranean (Verma et al. 2010). *L. angustifolia* EO (LAEO) primarily contains linalool and linalyl acetate (Caputo et al. 2016). Several studies have shown that *L. angustifolia* has a wide range of pharmacological effects (Verma et al. 2010), including antidiabetic, anxiolytic, antioxidant, antifungal, anti-inflammatory, sedative, carminative, antidepressant, and insect repellent properties (Caputo et al. 2016; Najibullah et al. 2021). Additionally, LAEO has shown activity against antibiotic-resistant bacteria, such as *methicillin*-resistant *Staphylococcus aureus* and *vancomycin*-resistant *enterococci* (Caputo et al. 2016).

In this context, the study aimed to identify potential volatile chemical compounds in LAEO that could contribute to its antidiabetic, antioxidant, dermatoprotective, neuroprotective, and antibacterial properties through in vitro methods. Additionally, molecular docking studies were employed to explore the interaction affinity between the major compounds identified and biological targets.

## Materials and methods

### Chemicals and reagents

TROLOX ((±)-6-Hydroxy-2,5,7,8-tetramethylchromane-2-carboxylic acid, 97%, a.g.), DPPH (2,2-Diphenyl-1-picrylhydrazyl, ACS), kojic acid (AS, > 99%), L(+)-ascorbic acid (> 99%, ACS), acarbose (> 95%), p-iodonitrotetrazoliumviolet (> 95%), 2,2-azino-bis-3-ethylbenzothiazoline-6-sulfonic acid (97%), Nitrophenyl-α-D–D-glucopyranoside (p-NPG) (> 98%), and l-3,4-dihydroxyphenylalanine (> 98% TLC), 5,5′-dithiobis(2-nitrobenzoic acid), 3,4-dihydroxy phenylalanine (L-DOPA) (> 98%), N-Succinyl-Ala-Ala-Ala-p-nitroanilide (97%) were obtained from Sigma Aldrich, USA. α-amylase solution (ex-porcine pancreas, EC 3.2.1.1) and α-glucosidase solution (from Saccharomyces cerevisiae, EC 3.2.1.20) were obtained from Sigma Aldrich, USA. Mueller–Hinton Agar, dimethyl sulfoxide (DMSO), and chloramphenicol were purchased from Biokar, Beauvais, France. All other reagents used were of analytical grade and obtained from reliable commercial sources.

### Plant collection and essential oil extraction

*L. angustifolia* Mill. was collected in its natural habitat, located in the Ouezzane province, northwest of Morocco, at coordinates 34° 47′ 50″ N and 5° 34′ 56″ W. The specimen, identified under the code RAB30, was authenticated by the Scientific Institute of Rabat (SIR) and preserved in the herbarium of the Moroccan Botany Department at the SIR. The samples were naturally air-dried in the shade at room temperature. EOs were extracted through hydrodistillation using a Clevenger-type apparatus. The extracted oils were quantified, dehydrated with anhydrous sodium sulfate, and stored at 4 °C for subsequent use.

### Chemical composition analysis

LAEOs were analyzed using GC–MS–MS following our previous study (Bouyahya et al. 2023). The procedure used a triple quadrupole tandem mass spectrometer linked to a gas chromatography TQ8040 NX (Shimadzu, Tokyo, Japan) system. Rxi-5 Sil MS column (30 m × 0.25 mm ID × 0.25 μm) capillary column and an apolar were employed for chromatography. The carrier gas used was pure helium. The injection volume was 1 µL. The source temperature was 200 °C. The chromatographic system was set up for splitless injections, which split opening at 4 min, a 250 °C injector temperature, and 37.1 kPa injector pressure. In this order, the temperature was planned to rise from 50 to 160 °C for 2 min, then to 280 °C for 2 min. Each product was identified by matching its mass spectra (MS) and retention index (RI), which were calculated using n-alkane series between C_8_ and C_20_, to those documented in the literature, as well as by computer matching with established reference databases (NIST version 2019).

### In vitro biological activities

#### Antidiabetic activity

##### α-amylase inhibitory assay

The potential inhibitory effect of LAEO on the α-amylase enzyme was evaluated by reacting varying quantities of the EO with α-amylase and a starch solution (Omari et al. 2023a, b). Specifically, 250 µL of the sample (from 50, 100, 150, 200, 250, and 300 µg/mL) were combined with 250 µL of α-amylase (240 U/mL) in a 0.02 M sodium phosphate buffer (pH = 6.9). The mixtures were incubated at 37 °C for 20 min. Subsequently, 250 µL of 1% starch solution in a 0.02 M sodium phosphate buffer (pH = 6.9) were added to the reaction mixture and incubated at 37 °C for 15 min. After adding 1 mL of dinitrosalicylic acid (DNS), the mixture was heated in a water bath for 10 min. The absorbance of the reaction mixture, diluted with 2 mL of distilled water, was measured at 540 nm using a UV-Vis spectrophotometer. To validate the accuracy of the results, acarbose, a well-established α-amylase inhibitor, was employed as a positive control.

The inhibition percentage was calculated using the following formula:

$$ \begin{gathered} \% {\text{ Inhibition}} \hfill \\ \;\; = [({\text{Abs}}_{{{\text{control}}}} - {\text{Ab}}_{{{\text{ssample}}}} )/{\text{Ab}}_{{{\text{scontrol}}}} ] \times 100 \hfill \\ \end{gathered} $$

The IC_50_ value was calculated by modeling the linear curve the percentage of inhibition versus the concentration of LAEO. It represents the concentration of the *α*-amylase inhibitor required to reduce 50% of the enzyme’s activity under the experimental conditions.

##### α-glucosidase inhibitory assay

Using the *p*-nitrophenyl α-d-glucopyranoside (pNPG) substrate, the α-glucosidase inhibitory activity of LAEO was determined following a modified version of the methodology reported in one of our previous studies (Omari et al. 2023a, b). Briefly, 200 µL of sample were mixed with 100 µL of 0.1 M sodium phosphate buffer (pH 6.7) containing 0.1 U/mL of α-glucosidase enzyme. The mixture was then incubated for 10 min at 37 °C. After this pre-incubation, 200 µL of a 1 mM pNPG solution in 0.1 M sodium phosphate buffer (pH 6.7) were added. The enzymatic reactions were then incubated for 30 min at 37 °C. The α-glucosidase activity was measured at a wavelength of 405 nm after adding 1 mL of 0.1 M Na_2_CO_3_ to the reaction solution. IC_50_ values were determined, and the inhibitory effect of LAEO was expressed as a percentage of inhibition. Acarbose was used as a positive control.

#### Antioxidant activity

##### DPPH assay

In this essay, the 2,2-diphenyl-1-picrylhydrazyl (DPPH) radical was used to assess the free radical scavenging activity of LAEO (El Omari et al. 2024b). Aliquots (0.2 mL) of LAEO was dissolved in methanol were added to 1.8 mL of a 0.004% methanolic solution of DPPH and incubated in triplicate at room temperature for 30 min. Then, the absorbance was measured at 517 nm using a UV spectrophotometer, against a blank. The percentage (%) to scavenge the DPPH radical was calculated according to the following formula:

DPPH scavenging activity (AA in %) = $\:\left(\frac{\left(\text{A}\text{c}-\:\text{A}\text{t}\right)}{\text{A}\text{c}}\right)$ × 100; where A_c_ is the absorbance of the control (without oil) and A_t_ is the absorbance of the sample (with oil).

Trolox was used as a positive control and the LAEO concentrations required to achieve 50% inhibition (IC_50_) were calculated by plotting the percentage inhibition against the sample concentrations. The assay was performed in triplicate and IC_50_ values ​​were reported as mean ± standard deviation (SD).

##### Ferric reducing power assay

Ferric Reducing Power was tested using our previously published method (El Omari et al. 2024b). Indeed, LAEO and the control were mixed with 2.5 mL of phosphate buffer (0.2 M, pH 6.6) and 2.5 mL of potassium ferricyanide [K3Fe(CN)6] (1%). The mixture was incubated at 50 °C for 20 min. Then, 2.5 mL of trichloroacetic acid (10%) was added to the mixture, which was centrifuged for 10 min at 3000 rpm. Subsequently, the upper layer of solution (2.5 ml) was mixed with distilled water (2.5 mL) and FeCl3 (0.5 ml, 0.1%), and the absorbance was measured at 700 nm using a spectrophotometer. The sample concentration providing 0.5 absorbance (IC_50_) was calculated by plotting the absorbance at 700 nm against the corresponding sample concentration. Trolox was used as a positive control. The assay was performed in triplicate and IC50 values were reported as mean ± SD.

#### ABTS radical scavenging activity

The ABTS (2,2′-azino-bis(3-ethylbenzothiazoline-6-sulfonic acid) cation radical was formed by mixing the ABTS stock solution (5 mL) with 5mL of potassium persulfate (K_2_S_2_O_8_) solution at 2.45 mM, stored in the dark at room temperature for 16 h before use (El Omari et al. 2024b). This solution was diluted with water to obtain an absorbance of 0.700 ± 0.015 at 734 nm and equilibrated at 30 °C. LAEA at various concentrations were diluted with DMSO to obtain sample solution. Then, 2.5 mL of sample solution was homogenized with 97.5 mL of ABTS solution. The mixture was incubated at room temperature for 6 min, and the absorbance was recorded at 734 nm. Blank samples were also prepared for each assay. Percentage inhibition was calculated using the following formula:

ABTS scavenging activity (%) = $\:\left(\frac{\left(\text{A}0-\:\text{A}1\right)}{\text{A}0}\right)$ × 100; Where A_0_ is the absorbance of the control and A_1_ the absorbance of the sample.

#### Dermatoprotective activity

##### Tyrosinase inhibitory assay

The dermatoprotective activity of LAEO was assessed by measuring tyrosinase inhibitory effect, following the procedure outlined by Marmouzi et al. (2019). In brief, 100 µL of tyrosinase solution (333 U/mL, 50 mM phosphate buffer, pH 6.5) were mixed with 25 µL of the sample and incubated for 10 min at 37 °C. Then, 300 µL of _L_-DOPA (5 mM) were added, and the mixture was incubated for an additional 30 min at 37 °C. Absorbance was recorded at 510 nm using a spectrophotometer. Tyrosinase inhibition was calculated at LAEO concentrations of 40, 60, 120, and 160 µg/mL, and IC_50_ values were determined. Quercetin was used as the positive control.

##### Elastase inhibitory assay

To assess the inhibitory effect of LAEO on elastase activity, a crucial enzyme involved in the degradation of elastin in blood vessels and skin, we followed the protocol outlined by Jeddi et al. (2023), With some modifications, the experiment involved dissolving varying concentrations of carvone, limonene, eucalyptol, α-terpineol, and *β*-caryophyllene were dissolved in methanol (0.5, 1, 2, and 3 mg/mL). Next, 200 µL of elastase solution prepared in Tris-HCl buffer (0.2 M, pH 8.0) was mixed with 50 µL of the sample. Following a 15-min incubation at 25 °C, 200 µL of *N*-succinyl-Ala-Ala-*p*-nitroanilide solution was added, and the reaction mixtures were thoroughly homogenized. Absorbance was then measured at 410 nm after an additional 20-min incubation at 25 °C. This method was used to determine the sample concentration needed to achieve 50% enzyme inhibition (IC_50_) and to calculate the percentage of elastase inhibition. To ensure the reliability of the experimental design, quercetin was included as a positive control.

#### Neuroprotective activity

The enzyme inhibition assay was performed following the procedure described by (Peron et al. 2024). The reaction mixture was prepared by combining 50 µL of the test fraction with 125 µL of DTNB (3 mM) and 25 µL of enzyme solution (AChE or BChE) in Tris-HCl buffer (pH 8.0), after an initial incubation at 25 °C for 15 min. This mixture was subsequently added to the substrate solution, consisting of 25 µL of either acetylthiocholine iodide (ATCI, 15 mM) or butyrylthiocholine chloride (BTCl, 1.5 mM). A control was prepared under identical conditions but without the test extract. After a further incubation of 15 min, absorbance was recorded at 405 nm. The inhibitory activities of AChE and BChE were expressed as galantamine equivalents (GALAE/g extract).

#### Antibacterial activity

##### Bacterial strains

To evaluate the antibacterial activity of LAEO, four bacterial strains were selected, including both Gram-negative and Gram-positive species: *Escherichia coli* ATCC 25,922, *Staphylococcus aureus* ATCC 29,213, *Pseudomonas aeruginosa* IH, and *Listeria monocytogenes* ATCC 13,932.

##### MIC and MBC determination

As described in previous studies, the minimum inhibitory concentration (MIC) was determined using the broth microdilution method (El Omari et al. 2023a, b). The minimum bactericidal concentration (MBC) is defined as the lowest concentration of the sample required to kill 99.9% of the bacterial population. The microdilution procedure used for MIC determination was also applied here. After 24 h of incubation at 37 °C, 10 µL of samples from tubes showing no visible growth were streaked onto Tryptone Soy agar (Biokar, Beauvais, France). The MBC was defined as the lowest concentration at which no growth was observed in the medium.

### In-silico biological activities

#### Ligand-receptor docking and complex visualization

The crystalline structures of α-amylase (PDB ID: 1HNY), α-glucosidase (PDB ID: 3TOP), AChE (PDB ID: 1ACL), and BChE (PDB ID: 1P0I) were sourced from the Protein Data Bank. Homology models for human tyrosinase and elastase were constructed using the crystal structure of mushroom tyrosinase (PDB ID: 2Y9X) and the protein structure of porcine pancreatic elastase (PDB ID: 4YM9) as templates. All 3D ligand structures were downloaded from the PubChem database. Molecular docking simulations were conducted using AutoDock Vina. Hydrogenation, partial charge distribution, and merging of non-polar hydrogen atoms were carried out with Autodocktools-1.5.7. Grid boxes were set up for each enzyme: a 40 Å x 16 Å x 60 Å box centered at X = 3.889, Y = 49.335, and Z = 22.552 for *α*-amylase; a 76 Å x 70 Å x 60 Å box centered at X = − 33.667, Y = 32.694, and Z = 27.889 for *α*-glucosidase; a 52 Å x 34 Å x 50 Å box centered at X = 2.474, Y = 68.367, and Z = 65.993 for AChE; a 44 Å x 32 Å x 28 Å box centered at X = 135.559, Y = 124.939, and Z = 55.848 for BChE; a 28 Å x 40 Å x 30 Å box centered at X = 11.03, Y = 8.605, and Z = 1.208 for elastase; and a 40 Å x 46 Å x 28 Å box centered at X = − 8.898, Y = − 29.357, and Z = − 46.526 for tyrosinase. The graphical visualization of all complexes was performed using Discovery Studio 2024 Client.

### Statistical analyses

Antioxidant, antidiabetic, and dermatoprotective tests were performed in triplicate to ensure repeatability and reliability of the results. Data were expressed as mean values ± standard error of the mean (SEM). Statistical analyses were carried out using GraphPad Prism 9, after calculation of the mean and standard deviation from triplicate experiments, with a two-way ANOVA followed by Bonferroni’s multiple comparison test. For the neuroprotective activity, an unpaired t-test was performed using the same software.

## Results

### Chemical composition

The GC–MS analysis of LAEO revealed 23 compounds (Table 1), accounting for 99.89% of the oil. Among these, oxygenated monoterpenes accounted for 82.32%, oxygenated sesquiterpenes for 7.35%, monoterpene hydrocarbons for 4.78%, and sesquiterpene hydrocarbons for 1.99%. The main components included linalyl acetate (31.78%), linalool (16.58%), limonene oxide (8.48%), deca-2,4-dienal (5.84%), and furanoid (5.25%). Additionally, minor amounts of fatty acids such as 9,12-octadecadienoic acid (Z, Z)- (0.54%) and oleic acid (1.54%) were detected.

**Table 1:** GC–MS–MS of LAEO

Number	Compound	*RI	Area
1	Beta-Myrcene	1166	1.89
2	p-Cymene	1205	0.58
3	Beta-Ocimene	1213	0.62
4	Ocimene	1220	1.13
5	Furanoid	1245	5.25
6	Linalool	1253	16.58
7	α-Terpineol	1334	3.07
8	Linalyl acetate	1459	31.78
9	(E, Z)-2,4-Decadienal	1509	3.38
10	Thymol	1510	0.98
11	Deca-2,4-dienal	1515	5.84
12	8-Hydroxylinalool	1518	0.7
13	limonene oxide	1522	8.48
14	2-Ethylhex-2-en-1-ol	1524	1.37
15	Neryl Acetate	1525	2.19
16	Geranyl acetate	1530	4.07
17	Copaene	1531	1.99
18	Neoalloocimene	1534	0.56
19	Spathulenol	1583	2.43
20	Caryophyllene oxide	1585	3.9
21	(1R,7 S, E)-7-Isopropyl-4,10-dimethylenecyclodec-5-enol	1594	1.02
22	9,12-Octadecadienoic acid (Z, Z)-	2069	0.54
23	Oleic acid	2175	1.54
Total			99.89
Monoterpene hydrocarbon			4.78
Sesquiterpene hydrocarbon			1.99
Oxygenated monoterpenes			82.32
Oxygenated Sesquiterpenes			7.35
Others			3.45

*RI: identification by Kovats indices. Retention index relative to C_6_–C_20_ on Rxi-5 Sil MS column

### Antioxidant activity

**Fig. 1:**
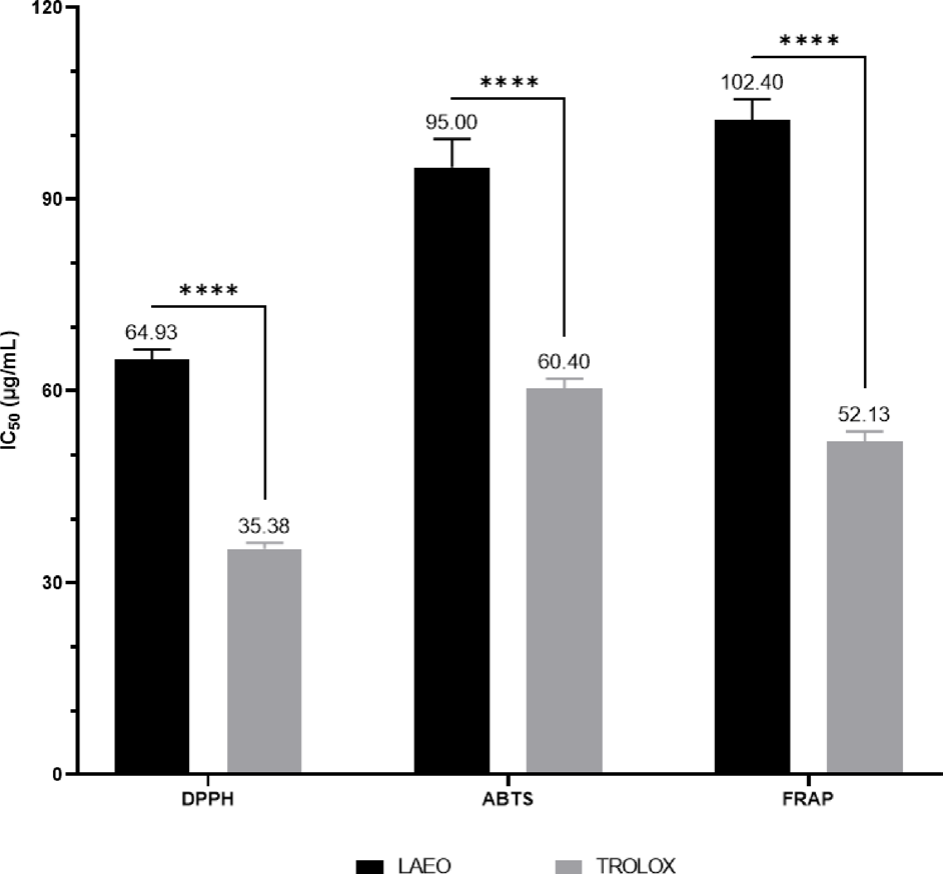
IC_50_ values (µg/mL) for the antioxidant activity of LAEO versus TROLOX

This study assessed the antioxidant activity of LAEO using three in vitro assays: DPPH, FRAP, and ABTS (Table 2; Fig. 1) expressed as IC₅₀ in µg/mL, The EO did not show significant antioxidant compared to Trolox. The EOs were able to reduce the stable DPPH free radical with an IC_50_ of 64.93 ± 1.62 µg/mL, showing weak antioxidant activity than Trolox (IC_50_ = 35.38 ± 0.91 µg/mL). The reducing power FRAP and ABTS assays followed the same trend as the DPPH tests but with less efficiency, with IC_50_ values of 120.40 ± 3.29 and 95.00 ± 4.45 µg/mL for FRAP and ABTS, respectively, compared to the synthetic antioxidant Trolox, which had IC_50_ values of 52.13 ± 1.56 and 60.40 ± 1.55 µg/mL in the FRAP and ABTS tests, respectively.

**Table 2:** Antioxidant properties (IC_50_ in µg/mL) of LAEO

	DPPH	ABTS	FRAP
LAEO	64. 93 ± 1.62 ^a^	95.00 ± 4.45 ^c^	102.40 ± 3.29 ^b^
TROLOX	35.38 ± 0.91 ^b^	60.40 ± 1.55 ^d^	52.13 ± 1.56 ^a^

Data with different superscript letters (in the same row) were significantly different (*p* < 0.05)

### Antibacterial effects

Our study assessed the antibacterial potential of LAEO on four multi-resistant bacterial strains: *L. monocytogenes*, *S. aureus*, *P. aeruginosa*, and *E. coli*, by determining the MIC and MBC (Table 3). According to our results, LAEO exhibited antibacterial effects against all four tested strains. Overall, the recorded MIC values were lower for Gram-positive bacteria than for Gram-negative bacteria, indicating a higher antibacterial potency against Gram-positive species. The lowest MIC value (0.0625% v/v) was observed against *L. monocytogenes*, followed by *S. aureus and P. aeruginosa* (both with a MIC of 0.125% v/v), and *E. coli* (MIC = 0.25% v/v). Additionally, the MBC/MIC ratio was equal to 1 when LAEO was tested against *S. aureus*, suggesting a bactericidal effect. In contrast, only a bacteriostatic effect was observed against the other three strains.

**Table 3:** The MIC and MBC values of LAEO expressed in % *v*/*v*

Molecules		Gram negative species		Gram positive species	
		*E. coli*	*P. aeruginosa*	*S. aureus*	*L. monocytogenes*
LAEO (%)	MIC	0.25	0.125	0.125	0.0625
	MBC	0.5	0.5	0.125	0.125
	MBC/MIC	2	4	1	2
Chloramphenicol	MIC	8	64	8	16
	MBC	32	64	32	32

### Enzymes inhibitory effects

#### Antidiabetic effects

**Fig. 2:**
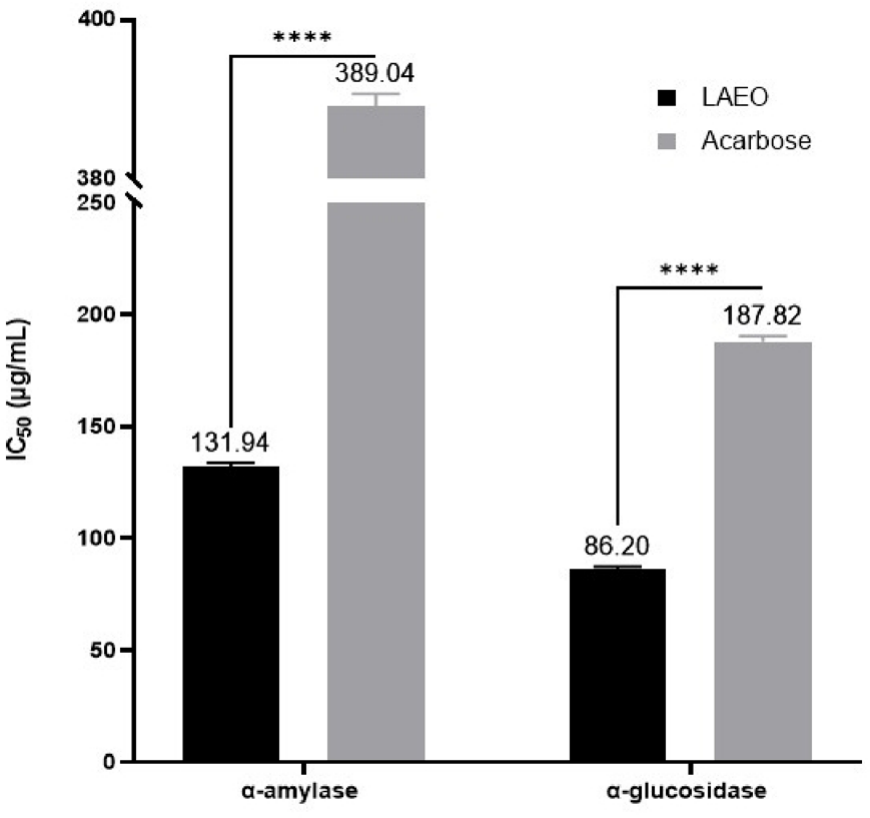
IC_50_ values (µg/mL) for the antidiabetic activity of LAEO versus Acarbose

The antidiabetic activity of LAEO was evaluated via α-amylase and α‐glucosidase inhibition assays (Table 4; Fig. 2). The results, expressed as IC₅₀ (µg/mL), showed that LAEO exerts strong inhibitory activity against both enzymes, α-amylase and α-glucosidase, with IC_50_ values of 131.94 ± 1.82 and 86.20 ± 1.23 µg/mL, respectively. For comparison, acarbose, had IC₅₀ values of 389.04 ± 1.65 µg/mL (α‐amylase) and 187.82 ± 2.62 µg/mL (α‐glucosidase). These data demonstrate that LAEO inhibits both enzymes more effectively than acarbose.

**Table 4:** Enzymatic inhibitory activity of LAEO

	Antidiabetic		Dermatoprotective		Neuroprotective	
	α-amylase (IC_50_ as µg/mL)	α-glucosidase (IC_50_ as µg/mL)	Tyrosinase (IC_50_ as µg/mL)	Elastase (IC_50_ as µg/mL)	AChE (mg GALAE/g)	BChE (mg GALAE/g)
LAEO	131.94 ± 1.82 ^a^	86.20 ± 1.23 ^c^	140.93 ± 3.30 ^b^	24.26 ± 3.94 ^d^	13.28 ± 1.00 ^e^	6.16 ± 0.98 ^f^
Acarbose	389.04 ± 1.65 ^b^	187.82 ± 2.62 ^d^	–	–	–	–
Quercetin	–	–	242.99 ± 2.67 ^a^	11.81 ± 0.90 ^c^	–	–

GALAE: Galantamine equivalent

In each activity, data with different superscript letters (in the same row) were significantly different (*p* < 0.05)

### Dermatoprotective effects

**Fig. 3:**
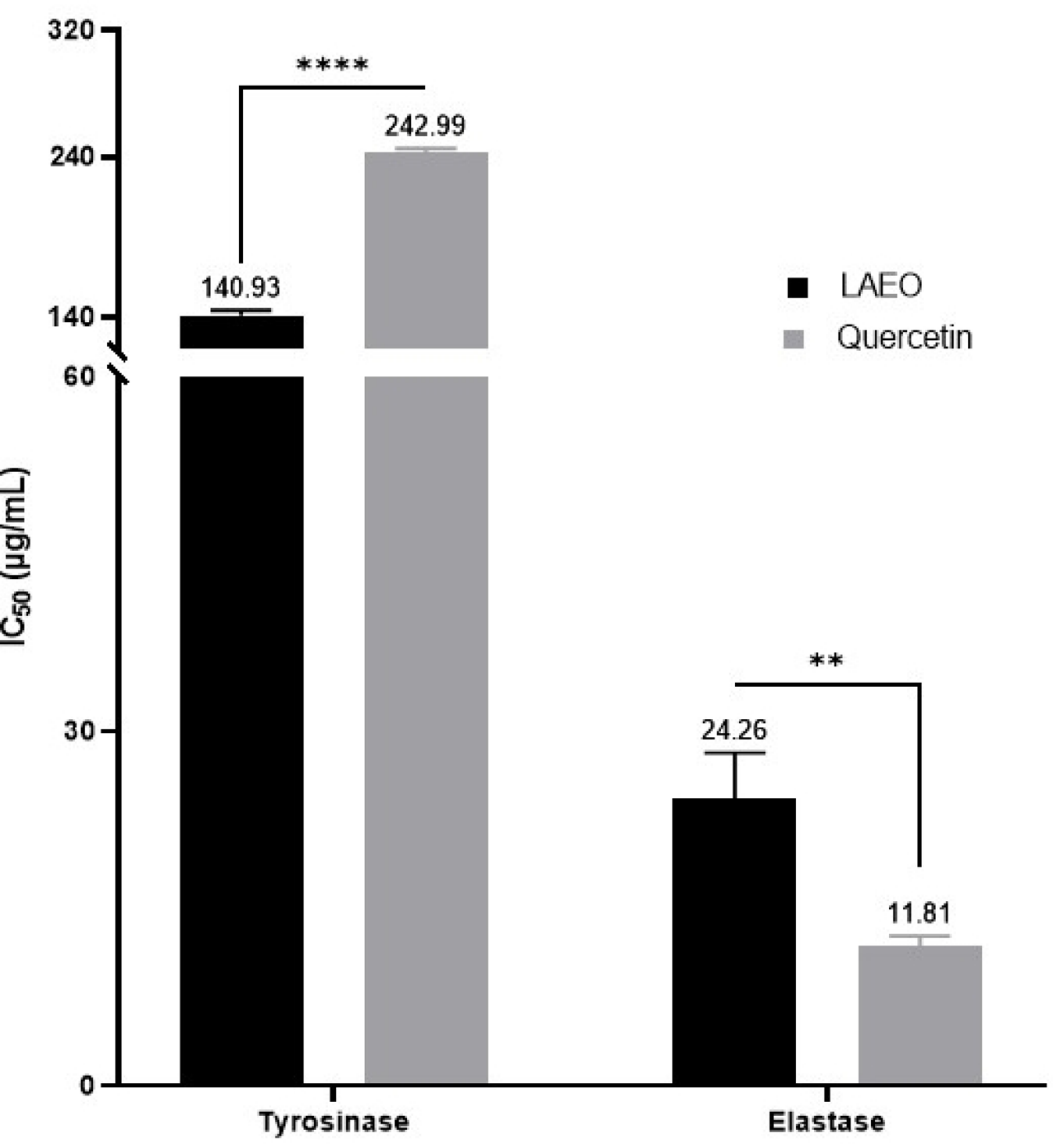
IC_50_ values (µg/mL) for the dermatoprotective activity of LAEO versus Quercetin

To evaluate the dermatoprotective properties of LAEO, its inhibitory activity against the enzymes tyrosinase and elastase was assessed. The results, expressed as IC₅₀ values and presented in Table 4; Fig. 3, revealed that LAEO inhibited tyrosinase with an IC₅₀ of 140.93 ± 3.30 µg/mL, whereas quercetin exhibited an IC₅₀ of 242.99 ± 2.67 µg/mL under the same conditions. In the elastase assay, LAEO’s IC₅₀ was 24.26 ± 3.94 µg/mL compared to quercetin’s IC₅₀ of 11.81 ± 0.90 µg/mL.

### Neuroprotective effects

**Fig. 4:**
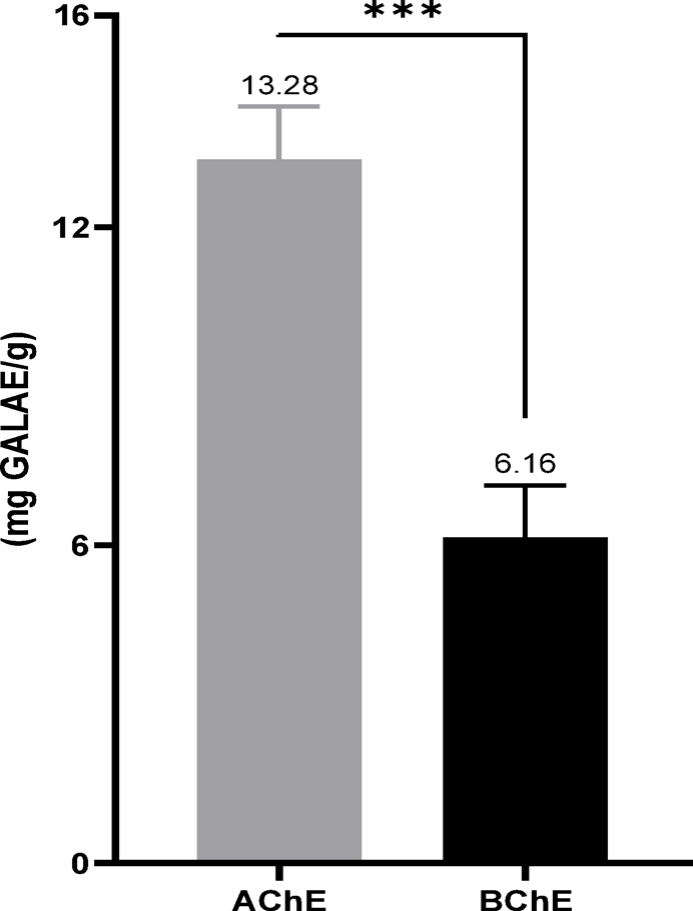
AChE and BChE inhibitory activity of LAEO in Galantamine equivalence

LAEOs have been studied for their inhibitory effect on AChE and BChE, with the results expressed in mg GALAE/g (Table 4; Fig. 4). As indicated, LAEO exhibited remarkable anti-cholinesterase enzymatic activities, with values of 13.28 ± 1.00 mg GALAE/g and 6.16 ± 0.98 mg GALAE/g for AChE and BChE, respectively.

### In silico biological activities

#### Molecular Docking

**Fig. 5:**
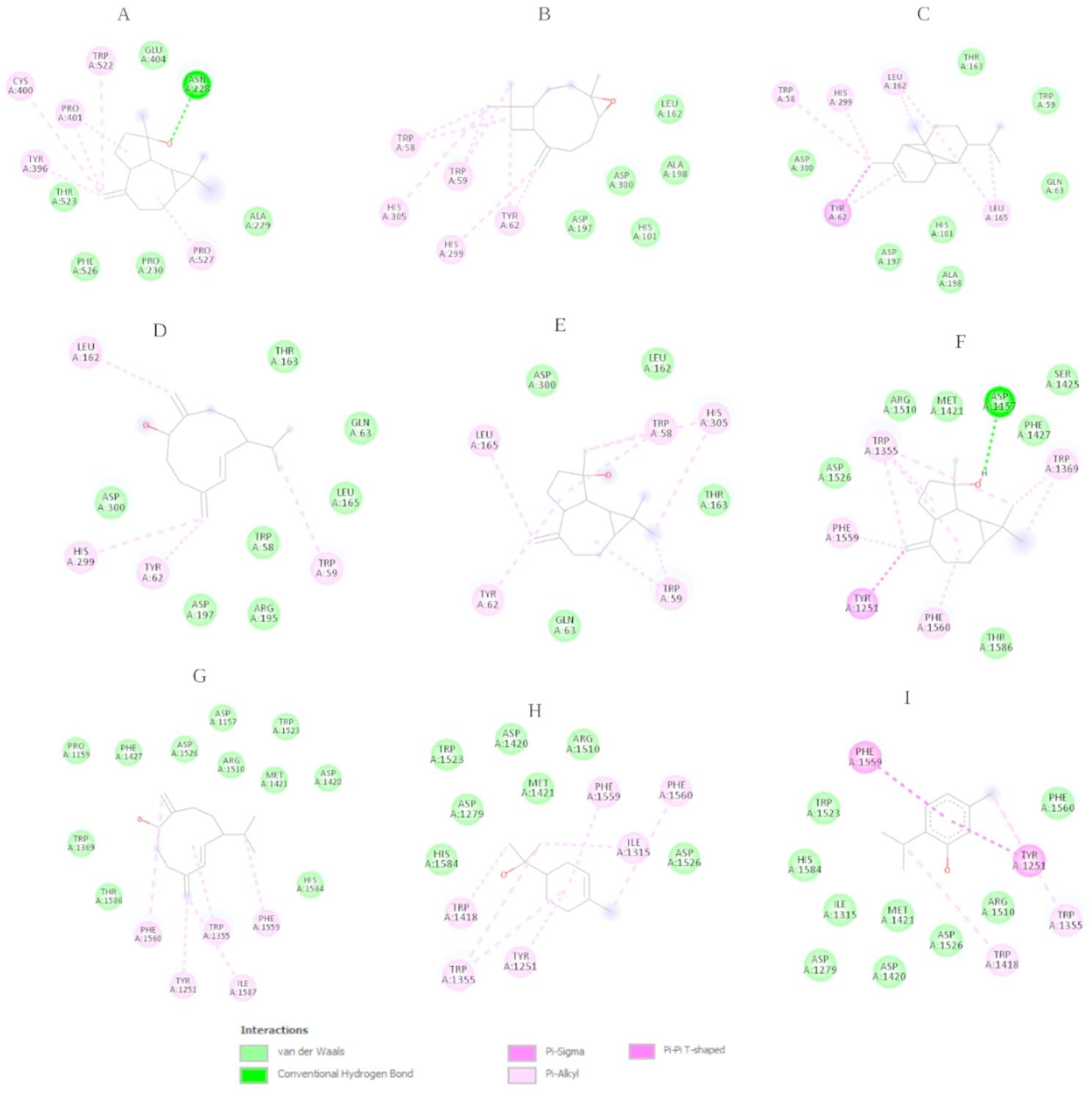
Representation of the 2D structure of Spathulenol – BChE (**A**), Caryophyllene oxide – α-amylase (**B**), (1R,7 S, E)-7-Isopropyl-4,10-dimethylenecyclodec-5-enol – α-amylase (**C**), Spathulenol - α-amylase (**D**), Spathulenol - α-glucosidase (**E**), (1R,7 S, E)-7-Isopropyl-4,10-dimethylenecyclodec-5-enol – α-glucosidase (**F**), α- terpineol - α-glucosidase (**G**) Thymol - α-glucosidase (H)

Molecular docking simulations were conducted to assess the binding affinities of 23 compounds identified in the LAEO against six target enzymes: AChE, BChE, α-glucosidase, α-amylase, tyrosinase, and elastase. Reference ligands used for comparison included galantamine, acarbose and quercetin. The docking results revealed a broad spectrum of binding affinities, with several compounds displaying notable interactions. Among these, spathulenol demonstrated the most consistent and potent activity across the majority of targets, achieving docking scores of − 8.9 kcal/mol against AChE, − 7.6 kcal/mol against BChE, − 7.2 kcal/mol against α-glucosidase, and − 6.7 kcl/mol against α-amylase. These values are comparable to those of the standard ligands, highlighting its strong binding potential. The 2D interaction map of the spathulenol–BChE complex (Fig. 5A) revealed a key hydrogen bond with ASN228, surrounded by multiple van der Waals and hydrophobic contacts with residues suggesting a stable and specific binding conformation. Similarly, in the α-amylase complex (Fig. 5D) and α-glucosidase (Fig. 5E) complex spathulenol engaged in extensive hydrophobic interactions with residues like TYR62, LEU162 and TRP59.

Other compounds, including caryophyllene oxide (–8.5 kcal/mol AChE, − 7.3 kcal/mol α-amylase), copaene (–8.7 kcal/mol AChE), and (1R,7 S, E)-7-isopropyl-4,10-dimethylenecyclodec-5-enol (–8.7 kcal/mol AChE, − 7.2 kcal/mol α-glucosidase), also showed high docking scores, particularly against cholinesterases and digestive enzymes. Their 2D interaction plots revealed a combination of *π*-alkyl, *π–π* stacking, and in some cases hydrogen bonding, reinforcing their favorable binding orientations within the active site pockets.

By contrast, the majority of LAEO compounds displayed moderate to low affinities for tyrosinase and elastase, with binding energies generally not exceeding − 6.3 kcal/mol. These findings indicate that while LAEO may not be particularly effective against tyrosinase or elastase, it contains several bioactive constituents, particularly spathulenol, that exhibit strong predicted interactions with key enzymes implicated in neurodegenerative and metabolic disorders (Table 5).

**Table 5:** Binding affinities of LAEO compounds against α-Amylase, α-Glucosidase, Elastase, Tyrosinase, AChE, and BChE protein structures (kcal/mol)

	Tyrosinase	Elastase		AChE		BChE	α-glucosidase	α-amylase
β-Myrcene	− 5.0	− 4.4		− 6.5		− 5.1	− 5.9	− 5.1
*p*-Cymene	− 5.3	− 4.7		− 7.1		− 6.0	− 6.6	− 5.8
β-Ocimene	− 5.3	− 4.4		− 6.8		− 5.2	− 6.2	− 5.1
Ocimene	− 5.2	− 4.3		− 6.7		− 5.3	− 6.4	− 5.1
Furanoid	− 5.3	− 4.9		− 6.7		− 5.9	− 5.5	− 5.5
Linalool	− 5.2	− 4.4		− 6.2		− 5.3	− 6.1	− 5.0
α-Terpineol	− 5.2	− 4.7		− 6.9		− 6.1	− 7.0	− 5.7
Linalyl acetate	− 5.1	− 5.0		− 7.1		− 5.7	− 5.5	− 5.7
(E.Z)-2.4-Decadienal	− 5.6	− 4.4		− 5.9		− 5.0	− 5.8	− 4.8
Thymol	− 5.8	− 4.8		− 7.2		− 5.8	− 7.0	− 5.9
Deca-2.4-dienal	− 4.7	− 4.4		− 6.0		− 5.0	− 5.4	− 4.7
Neoalloocimene	− 5.4	− 4.5		− 6.8		− 5.3	− 6.1	− 5.7
8-Hydroxylinalool	− 5.0	− 4.6		− 6.4		− 5.1	− 6.1	− 5.4
Limonene oxide	− 5.3	− 4.7		− 7.0		− 6.0	− 6.4	− 5.6
2-Ethylhex-2-en-1-ol	− 4.9	− 3.7		− 5.6		− 4.6	− 5.3	− 4.6
Neryl Acetate	− 5.6	− 5.1		− 7.1		− 5.3	− 6.6	− 5.3
Geranyl acetate	− 5.4	− 5.0		− 7.0		− 5.7	− 6.4	− 5.5
Copaene	− 6.3	− 5.5		− 8.7		− 6.8	− 6.7	− 7.0
Spathulenol	− 6.1	− 5.5		− 8.9		− 7.6	− 7.2	− 6.7
Caryophyllene oxide	− 6.0	− 5.5		− 8.5		− 7.2	− 6.3	− 7.3
(1R.7 S.E)-7-Isopropyl-4.10-dimethylenecyclodec-5-enol	− 5.4	− 5.6		− 8.7		− 7.0	− 7.2	− 6.8
9.12-Octadecadienoic acid (Z.Z)-	− 4.9	− 4.4		− 7.2		− 6.3	− 6.1	− 5.4
Oleic acid	− 4.6	− 4.6		− 6.9		− 5.7	− 6.0	− 5.2
Standards	Quercetin	− 7.7	− 6.7		–	–	–	–
	Galantamine	–	–		− 9.2	− 7.7	–	–
	Acarbose	–	–		–	–	− 7.1	− 6.4

## Discussion

The chemical composition of LAEO reveals a rich mixture of bioactive compounds, notably dominated by linalyl acetate (31.78%) and linalool (16.58%), limonene oxide (8.48%) and furanoid (5.25%). When compared with studies from other regions of Morocco, both similarities and differences are observed. For instance, LAEO from Azrou (Radi et al. 2021), contained higher amounts of camphor (20.07%) and 1,8-cineole (16.68%), with lower levels of linalyl acetate (13.66%) and linalool (12.34%). Oils from Essaouira and Marrakech-Safi (Moussi Imane et al. 2017a), also showed significant levels of 1,8-cineole (18.07%), camphor (11.89%), and borneol (8.47%), while maintaining notable linalool and linalyl acetate contents. In contrast, LAEO from India (Verma et al. 2010), was composed mainly of linalyl acetate (47.56%) and linalool (28.06%), a pattern also seen in oils from Poland (Smigielski et al. 2018), Crimea (Białoń et al. 2019), and Australia (Shellie et al. 2002), though with varying proportions. On the other hand, LAEO from Iran (Bakhsha et al. 2014), exhibited a completely different profile, dominated by carvacrol (26.2%), limonene (19.6%), and 1,8-cineole (11.8%), indicating the strong influence of environmental and geographical factors on oil composition. These differences are not merely chemical but also biological, as the efficacy and application of LAEO are closely tied to its constituent profile. Oils rich in linalool and linalyl acetate, are widely recognized as high-quality oils (Peana et al. 2002), particularly valued in the aromatherapy and cosmetic industries due to their pleasant aroma and multiple therapeutic properties (Woronuk et al. 2011).

Despite this promising chemical richness, LAEO demonstrated weak antioxidant activity in vitro, as evaluated by DPPH, ABTS, and FRAP assays, compared to the synthetic antioxidant Trolox. This was clearly reflected in the significantly higher IC₅₀ values recorded for the oil, indicating a reduced ability to effectively scavenge free radicals or reduce oxidized molecules. Such diminished antioxidant capacity is likely due to the low content of phenolic compounds in the EO, as these compounds are well-documented for their ability to neutralize reactive oxygen species and contribute strongly to antioxidant activity (Miguel 2010). Supporting this observation, a study examining *L. angustifolia* extracts obtained through polar solvents such as water and ethanol reported substantially stronger antioxidant activity in these extracts. This enhanced performance was directly associated with their higher levels of polyphenolic compounds, with total polyphenol contents of 45.30 mg GAE/g dry extract for the water extract and 14.40 mg GAE/g for the ethanol extract (Talić et al. 2023a). Furthermore, additional studies have also reported similar weak antioxidant effects for LAEO, reinforcing our findings (Bevanda; Talić et al. 2023a). This may suggest that phenolic content plays an important role in determining antioxidant potential. Additionally, the extraction method could also contribute to these differences (Celiktas et al. 2007).

LAEO demonstrated a stronger antibacterial effect, consistent with prior studies showing Gram-negative bacteria are generally less sensitive to EOs than Gram-positive bacteria. This resistance in Gram-negative bacteria is due to their outer membrane, which restricts hydrophobic substances via a lipopolysaccharide coating. In contrast, Gram-positive bacteria lack this barrier, allowing hydrophobic EO components to interact directly with their cell membranes, increasing permeability and disrupting enzymatic systems (Burt 2004; Džamić et al. 2015; El Omari et al. 2024a, b; Habtemariam 2016; Nada et al. 2003).

Many studies have confirmed the potent antibacterial effects of LAEO against various pathogens, including the four species studied in this study. For example, LAEO from the Taounate region of Morocco showed significant activity against *S. aureus*, *E. coli*, *Bacillus cereus*, *Micrococcus luteus*, *Salmonella enterica*, and *Klebsiella aerogenes*, with MIC and MBC values from 0.125 to 1.0% (v/v), indicating bactericidal action. Gram-positive strains were more sensitive than Gram-negative ones, corroborating our findings (Jeddi et al. 2023).

Similarly, LAEO from Essaouira, Morocco exhibited strong antibacterial activity against *S. aureus* ATCC 25,923, *E. coli* ATCC 25,922, and *P. aeruginosa* ATCC 27,853, with MIC values of 1.33, 3.33, and 42.67 µL/mL, respectively. This study suggested a bactericidal effect against *P. aeruginosa* and *E. coli*, and a bacteriostatic effect against *S. aureus* (Moussi Imane et al. 2017a, b). Hussain et al. (2011) also reported greater sensitivity of Gram-positive strains such as *S. aureus*, *B. cereus*, *B. subtilis*, and *B. pumilus* to LAEO compared to Gram-negative strains like *P. aeruginosa*, *E. coli*, and *S. poona*, aligning with our results.

Adaszyńska-Skwirzyńska & Szczerbińska (2018) found Polish LAEO most effective against *S. aureus* ATCC 25,923 (MIC 0.25% v/v), followed by *E. coli* ATCC 25,922 (0.625% v/v), and *P. aeruginosa* (2% v/v). Tuscan (Italy) LAEOs tested against *E. coli* showed MIC and MBC values of 3.13% v/v, confirming bactericidal effects (Garzoli et al. 2021).

In Australia, LAEO was highly effective against *S. aureus* (Gram-positive), but P. *aeruginosa* (Gram-negative) was unaffected, with only moderate activity against *E. coli* (Danh et al. 2013). Another study reported the strongest inhibition zone for *S. aureus* ATCC 6538 (24 mm), followed by MRSA 15 (18.3 mm) and *E. coli* DSM 1077 (13.66 mm), confirming the lower sensitivity of Gram-negative bacteria (El Kharraf et al. 2021). Smigielski et al. (2018) found higher antibacterial activity against *B. subtilis* ATCC 6633 and *S. aureus* ATCC 1803 (MIC 0.4–0.9 µg/mL) compared to *E. coli* and *P. aeruginosa* (MIC 0.9–1.9 µg/mL). Niksic et al. (2017) demonstrated LAEO’s efficacy against *S. aureus* and *P. aeruginosa* but no activity on *E. coli* at 5% concentration. Tardugno et al. (2019) identified *L. monocytogenes* strains as highly sensitive to LAEO, supporting the trend of Gram-positive sensitivity.

Blažeković et al. (2018) evaluated the antibacterial activities of LAEO were assessed against 14 clinically relevant bacteria and common contaminants in food, cosmetics, and pharmaceuticals. Gram-positive bacteria tested included *B. cereus*, *B. pumilus*, *Enterococcus faecalis*, *Kocuria rhizophila*, *L. monocytogenes*, *S. aureus*, and *Streptococcus pyogenes*, while Gram-negative species included *E. coli*, *Klebsiella oxytoca*, *Klebsiella pneumoniae*, *Proteus mirabilis*, *P. aeruginosa*, *Salmonella enteritidis*, and *Yersinia enterocolitica*. Overall, Gram-positive species had larger growth inhibition zones (14–19 mm) and greater sensitivity than Gram-negatives (10–17 mm), with *S. pyogenes* showing the highest sensitivity and *P. mirabilis* the lowest (Blažeković et al. 2018).

When combined with antibiotics like chloramphenicol, ciprofloxacin, nystatin, and fusidic acid, LAEO showed significant synergistic effects, particularly with chloramphenicol against *P. aeruginosa* (ΣFIC 0.29) and ciprofloxacin against *S. aureus* (ΣFIC 0.29). Synergy was most pronounced when larger amounts of LAEO were included in the mixtures (de Rapper et al. 2013). Bogdan et al. (2021) reported marked bactericidal effects of LAEO against *S. aureus* and *E. coli*, and mixtures of lavender and tea tree oils effectively halted bacterial growth. LAEO combined with *Artemisia herba-alba* and *Rosmarinus officinalis* also showed synergy at low concentrations (Messaoudi Moussii et al. 2020). Further evaluations involving LAEO with 45 other EOs revealed interactive properties, with the most favorable effects when combined with *Citrus sinensis* and higher amounts of *Daucus carota* EO (de Rapper et al. 2013).

*L. angustifolia* is traditionally used for treating staphylococcal infections like boils and abscesses (de Rapper et al. 2013). LAEO treatment was shown to modify macrophage responses to *S. aureus* by improving phagocytosis, controlling intracellular bacterial multiplication, suppressing pro-inflammatory cytokines, and stimulating heme oxygenase-1 gene transcription. These immunomodulatory effects suggest LAEO could help reduce inflammation and regulate immune responses in bacterial infections (Giovannini et al. 2016).

Gismondi et al. (2021) assessed LAEO’s antibiotic potential in hospital environments by vaporizing it in five areas of an Italian hospital over three months. They monitored contamination levels of *Staphylococcus* species and methicillin-resistant bacteria, finding LAEO reduced bacterial counts in all sections, with *S. epidermidis* reduced by up to 60.84% and *S. aureus* by 88.33%. Gismondi et al. (2021) similarly reported that EO blends containing 24% LAEO reduced hospital room bacterial contamination by 90%. These findings highlight LAEO’s strong, targeted bioactivity against environmental pathogens and support its use in public space disinfection systems.

Besides antibacterial activity, LAEO also exhibits enzyme inhibitory potential relevant to antidiabetic, dermatoprotective, and neuroprotective effects.

Among the important factors that can increase postprandial blood glucose levels is the breakdown of complex sugars into simple sugars (Bouyahya et al. 2021; Sarikurkcu et al. 2015). Numerous enzymes, such as α-amylase and α-glucosidase, contribute to regulating this process. Complex dietary carbohydrates are broken down into oligosaccharides and disaccharides by α-amylase, which are then converted into monosaccharides by α-glucosidase (Bouyahya et al. 2021; Koki et al. 2022). These enzymes hydrolyze carbohydrates into simple sugars such as glucose. Inhibition of α-glucosidase can delay carbohydrate digestion, resulting in a decrease in the rate of glucose absorption, which is a primary goal of antidiabetic treatment (Koki et al. 2022; McDougall et al. 2005). This inhibition is a key strategy in managing postprandial blood glucose levels (Dong et al. 2012; Sarikurkcu et al. 2015). However, A significant limitation of currently used α-amylase and α-glucosidase inhibitors (such as acarbose and viglib) is their adverse side effects (Nakhaee and Sanjari 2013), including flatulence, abdominal distension, and diarrhea (Bouyahya et al. 2021; Dong et al. 2012; El hassouni et al. 2021; Sarikurkcu et al. 2015). Our results show that LAEO significantly inhibits both α-amylase (IC₅₀ = 131.94 µg/mL) and α‐glucosidase (IC₅₀ = 86.20 µg/mL), outperforming acarbose in vitro. These findings are consistent with other studies on the ability of LAEO to manage metabolic disorders through its antihyperglycemic effect. Recently, LAEO from samples collected in the Taounate region of Morocco demonstrated significant inhibitory activity against α-amylase and α-glucosidase (*p* < 0.05) (Jeddi et al. 2023). In fact, LAEO inhibited α-amylase with an IC_50_ value of 3.20 mg/mL, comparable to that of acarbose (IC_50_ = 3.06 mg/mL). Additionally, at a concentration of 1 mg/mL, LAEO inhibited α-amylase by 84%, slightly outperforming acarbose, which inhibited it by 83%. LAEO also significantly inhibited *α*-glucosidase (IC_50_ = 2.81 mg/mL), comparable to the acarbose control (IC_50_ = 2.81 mg/mL). At a concentration of 1 mg/mL, LAEO showed an inhibition of α-amylase activity of 80.2%, compared to 86.16% for acarbose (Jeddi et al. 2023). The EO from *L. angustifolia* leaves in India also showed inhibition of α-glucosidase with an IC_50_ of 609.44 µg/mL, a value close to that of acarbose (IC_50_ = 526.5 µg/mL), aligning with our findings (Najibullah et al. 2021). El Kharraf et al. (2021) also found that LAEO, combined with *R. officinalis* and *Citrus aurantium* EO obtained by simultaneous steam distillation-hydrodistillation, exerted remarkable inhibitory capacity. Similarly, two methanolic extracts of *L. angustifolia* from Calabria (southern Italy) significantly inhibited α-glucosidase (2.1 and 2.5 mg/mL) and α-amylase (6.8 and 14.8 mg/mL) (Tundis et al. 2023).

LAEO has demonstrated significant inhibitory effects on α-glucosidase and α-amylase. By acting on these key digestive enzymes, LAEO presents a valuable approach for managing diabetes through the reduction of glucose absorption (Honda and Hara 1993).

The skin is the body’s largest organ, with a surface area of approximately 2 m² and a weight of about 5 kg (Dréno 2009; Gushiken et al. 2021). It comprises the epidermis, dermis, and hypodermis, located deeper. The epidermis is a stratified keratinized squamous epithelium, while the dermis is a fibrous-elastic tissue with blood vessels, nerves, and sensory receptors that support the skin. The hypodermis, the deepest layer, is primarily composed of adipose tissue pads and is attached to the deep fascia or periosteum (Arda et al. 2014; Manna et al. 2024).

Melanin is produced by melanocytes in the basal epidermis through melanogenesis (Costin and Hearing 2007; Kim and Uyama 2005; Rangkadilok et al. 2007; Wang et al. 2006). Melanocytes, about 8% of epidermal cells, originate from melanoblasts (Sviderskaya et al. 2001). Melanin, crucial for pigmentation and UV protection, varies with eumelanin and pheomelanin ratios and melanosome number (Schlessinger and James 2018). L-tyrosine promotes melanosome synthesis and tyrosinase activity, regulating melanogenic homeostasis (D’Ischia et al. 2013; Slominski et al. 2012).

Tyrosinase (EC 1.14.18.1) is the key enzyme in melanin biosynthesis, a copper-containing membrane glycoprotein in melanosomes (Mapunya et al. 2012; Panzella and Napolitano 2019). It acts on phenols and catechols to form ortho-quinones (Pillaiyar et al. 2017). Tyrosinase hyperactivity can cause skin issues like age spots and melanomas (Mapunya et al. 2012).

In our study, LAEO demonstrated stronger tyrosinase inhibition (IC₅₀ = 140.93 µg/mL) than quercetin (IC₅₀ = 242.99 µg/mL). Compared to other studies, this anti-tyrosinase activity is higher than that of rosemary EO (IC_50_ = 923 µg/mL) (Zhang et al. 2017), but lower than that of *Eucalyptus globulus* EO (IC_50_ = 38.21 ± 0.13 µg/mL) (Assaggaf et al. 2022) and coriander EO (IC_50_ = 34.14 ± 2.1 µg/mL), which showed greater inhibitory activity against tyrosinase. However, the anti-elastase effect of LAEO (IC_50_ = 24.2633 ± 3.1628 µg/mL) was less potent compared to quercetin (IC_50_ = 11.81 ± 0.736 µg/mL) (Fig. 3). Compared to other research, the elastase inhibitory activity of our sample was higher than that of *Mentha viridis* EO (IC_50_ = 114.24 ± 1.22 µg/mL) (Bouyahya et al. 2020) and *Ajuga iva* EO at two stages; vegetative (IC_50_ = 192.21 ± 7.80 µg/mL) and flowering (IC_50_ = 261.13 ± 1.72 µg/mL) (Bouyahya et al. 2021)These findings suggest that preparations based on *L. angustifolia* could be utilized not only in sunscreen formulations to protect against sunburn and slow skin aging but also in treatments for hyperpigmentation disorders commonly associated with aging (Al-Mijalli et al. 2022).

Our results confirm the neuroprotective potential of LAEO, as evidenced by its notable inhibitory activity against cholinesterase enzymes. This aligns well with previous findings, such as those by Talić et al. (2023b), who reported 39.5% inhibition of AChE at 1 mg/mL, and Sekeroglu et al. (2022), who observed even stronger inhibition rates of 81.62% for AChE and 84.12% for BChE at 800 µg/mL. Moreover, studies on Aβ-induced Alzheimer’s disease models in rats demonstrated that aqueous extracts of L. angustifolia improve memory and cognitive functions (Oskouie et al. 2018).

Phytochemical analyses showing linalyl acetate (31.78%) and linalool (16.58%) as major constituents suggest that these compounds likely contribute significantly to the observed enzyme inhibition. Supporting this, Bonesi et al. (2010) reported AChE and BChE inhibitory activities of linalyl acetate with IC_50_ values of 82 and 168.7 ± 2 µg/mL, respectively, while Mitsuo Miyazawa et al. (1998) demonstrated 38% AChE inhibition by this compound at 82 µg/mL. Additionally, numerous studies have confirmed the AChE inhibitory effects of linalool (Farag et al. 2016; Perry et al. 2000; Radwan et al. 2024; Xu et al. 2017).

Taken together, our findings strongly suggest that LAEO is a valuable natural source of bioactive molecules that could serve as effective active ingredients in medicinal products. Its potent inhibitory effect on AChE supports the hypothesis that LAEO may be developed as a natural therapeutic agent for the management of neurodegenerative disorders.

The molecular docking analysis of LAEO compounds revealed that several constituents exhibited promising inhibitory activities against key enzymes linked to neurodegenerative and metabolic disorders. Among the identified compounds, spathulenol stood out due to its strong binding affinities across several key biological targets, notably AChE, BChE, α-glucosidase, and α-amylase. These binding energies are comparable to or exceed those of standard therapeutic inhibitors, suggesting that spathulenol possesses substantial potential as a dual neuroprotective and antidiabetic agent.

Furthermore, the biological relevance of spathulenol is reinforced by experimental studies (Manjima et al. 2021). A notable investigation reported its neuroprotective efficacy in SH-SY5Y neuroblastoma cells exposed to 6-hydroxydopamine (6-OHDA), a model of Parkinson’s disease-associated neurotoxicity. In this study, spathulenol significantly reduced oxidative stress markers, preserved the mitochondrial membrane potential, and decreased apoptosis in a dose-dependent manner. These protective effects were attributed to its antioxidant activity, which mitigated the mitochondrial dysfunction and ROS overproduction commonly induced by 6-OHDA. This experimental evidence strongly supports the neurotherapeutic implications suggested by the molecular docking results.

In addition to its neuroprotective potential, spathulenol has been frequently cited in the literature for a broad range of bioactivities. Essential oils rich in spathulenol have demonstrated antibacterial (Terezinha de Oliveira et al. 2019), antifungal (Damian-Badillo et al. 2008), and anti-tumor (Bomfim et al. 2016) properties. It has also been explored for applications in diabetes management and skincare (Aljarah and Hameed 2018; Movahhedin et al. 2016). The many different health benefits linked to spathulenol show that it can act in several ways, making it an important compound in LAEO. Because of this, it deserves to be studied more closely for its potential medical use.

## Conclusion

This study highlights the rich phytochemical profile and promising bioactivities LAEO from the Ouezzane region, particularly its significant inhibitory effects on enzymes associated with antibacterial, antidiabetic, dermatoprotective, and neuroprotective activities. Among its constituents, spathulenol emerged as a key bioactive compound, demonstrating strong binding affinities with multiple therapeutic targets such as AChE, BChE, α-glucosidase, and α-amylase, suggesting its potential as a dual-action agent in managing neurodegenerative and metabolic disorders. We propose that future investigations focus on spathulenol through molecular dynamics simulations to evaluate the stability and behavior of its enzyme-ligand interactions in dynamic biological environments. Furthermore, we recommend exploring the underlying mechanisms of LAEO’s anti-tyrosinase activity, which may involve synergistic interactions between various oil constituents. Such studies, incorporating bio-guided fractionation and combination assays, could provide valuable insights for developing effective multi-target natural therapeutics.

Moroccan-cultivated LAEO emerges as a promising reservoir of bioactive potential, holding the key to the development of innovative natural therapeutics. While the findings illuminate its multifaceted biological properties, the journey toward its clinical application necessitates rigorousness in vivo studies and clinical trials to confirm efficacy and determine safe therapeutic thresholds.

## Data Availability

All data generated or analysed during this study are included in this published article.
